# Prenatal Diagnosis and Postnatal Followup of Partial Trisomy 13q and Partial Monosomy 10p: A Case Report and Review of the Literature

**DOI:** 10.1155/2012/821347

**Published:** 2012-10-23

**Authors:** Yuan Wei, Xuefeng Gao, Liying Yan, Fang Xu, Peining Li, Yangyu Zhao

**Affiliations:** ^1^Department of Obstetrics and Gynecology, Peking University Third Hospital, Beijing 100191, China; ^2^Laboratory of Molecular Cytogenetics and Genomics, Department of Genetics, Yale University School of Medicine, New Haven, CT 06520, USA

## Abstract

We report prenatal diagnosis and postnatal findings of a fetus with partial trisomy of 13q21.33-qter and partial monosomy of 10p15.3-pter. The mother is a known carrier of a balanced translocation, t(10;13)(p15.3;q21.33), ascertained by history of one miscarriage and two neonatal deaths. The fetal karyotyping on cultured amniocytes showed 46,XX,der(10)t(10;13)(p15.3;q21.33). Oligonucleotide array comparative genomic hybridization (aCGH) defined a 2.339 Mb distal deletion at 10p15.3 (chr10:126,161–2,465,089) and a 46.344 Mb duplication of 13q21.33–q34 (chr13:67,779,708–114,123,540). Ultrasound examination showed polydactyly and polyhydramnios in the fetus. After genetic counseling, the mother decided to continue the pregnancy, and follow-up ultrasound monitoring found no further abnormalities. A girl was delivered at 37^+6^ weeks of gestation and was transferred to the intensive care unit for intermittent convulsions within 26 hours. She was diagnosed with neonatal hypoxic ischemic encephalopathy and experienced several episodes of apnea in the following month. Her birth weight was 2900 g (10–25th centile) and at five months was 5500 g (5–10th centile). She had dysmorphic features and mild psychomotor retardation. A review of the literature found three previously reported cases with similar compound 10p/13q abnormalities. We discuss a two-step approach to assess fetal viability and phenotype using genomic information from partial trisomy and monosomy.

## 1. Introduction

Reciprocal translocation occurs with a frequency of 1 in every 500 liveborn infants [1]. Carriers of a balanced reciprocal translocation will produce genetically unbalanced gametes and lead to increasing risk of infertility, miscarriages, or deliver of abnormal offspring. Prenatal diagnosis is effective in detecting partial trisomy and partial monosomy resulting from a known carrier of a reciprocal translocation, but follow-up genetic counseling can be challenging due to concerns of fetal viability in later pregnancy and normality after birth. Current practice involves phenotype-genotype inference through the comparison of a detected compound abnormality with similar cases published in the literature. However, cases with similar compound abnormalities are rare, and usually the few cases reported by conventional cytogenetic analysis lack the genomic coordinates for gene content and postnatal followup if the pregnancy was terminated. Here, we report prenatal diagnosis of partial trisomy of 13q and partial monosomy of 10p in a fetus with polydactyly and polyhydramnios. Further analysis using oligonucleotide aCGH defined the genomic size and gene content for the 10p deletion and 13q duplication. Review of the literature found that there were three previously reported cases with compound 10p/13q abnormalities. Clinical findings from postnatal followup were compared with previous cases to evaluate the compound effect from this partial monosomy 10p and partial trisomy 13q.

## 2. Clinical Report

**Figure 1:**
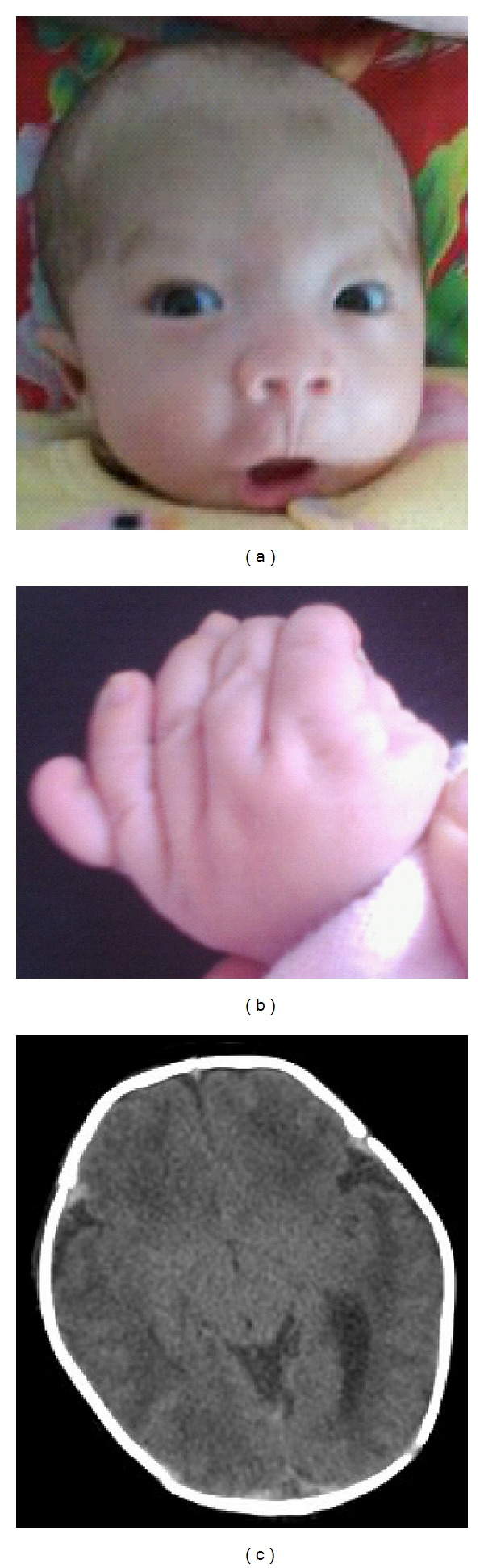
Photographs of patient at one-month old. (a) Facial features of frontal bossing, low and flat nasal bridge, low-set ears, nasal bridge hypoplasia, hypertelorism, and bilateral epicanthus. (b) Polydactyly of her left hand. (c) CT scan of subarachnoid hemorrhage.

A 32-year-old woman with a known balanced translocation between chromosomal bands 10p15.3 and 13q21.33 was referred for prenatal diagnosis and genetic counseling at 20 weeks gestation because of an ultrasound-detected fetal abnormality. This is her fourth pregnancy. Her first baby had hydrocephalus and cleft palate and died 3 days after birth. The second baby showed cleft palate and small short penis and died 2 days after birth. The third gestation terminated in spontaneous abortion 10 weeks after conception. Other family members or relatives were normal, and there was no family history of congenital malformations or other genetic disorders. Amniocentesis was performed and routine G-band analysis of cultured amniocytes showed a derivative chromosome 10 from maternal t(10;13) and a normal pair of chromosome 13. After genetic counseling, the couple chose to continue the pregnancy. Follow-up ultrasound monitoring found no more abnormalities. The fetus' heart was morphologically normal at fetal echocardiography. The fetal growth rate was normal for the gestational age. Spontaneous labor began at 37^+6^ gestation weeks. A girl was delivered with a birth weight of 2900 g (10–25th centile) and Apgar scores of 9 and 10 at 1 and 5 min, respectively. About 26 hours later, the newborn presented paroxysms of crying and convulsion. She was diagnosed with neonatal hypoxic ischemic encephalopathy and experienced apnea several times in the following month. The neonatal evaluation revealed some dysmorphic features including frontal bossing, low and flat nasal bridge, low-set ears, nasal bridge hypoplasia, hypertelorism, bilateral epicanthus, high-arched palate, short broad neck, thin upper lip, and polydactyly (6th finger at left hand) (Figure 1). Her weight was 5500 g at 5 months old (5–10th centile). She had mild psychomotor retardation.

## 3. Results of Cytogenomic Analysis

Genomic DNA was extracted from cultured amniocytes using the Gentra Puregene kit (Qiagen, Valencia, CA, USA). DNA concentration was measured using a NanoDrop spectrophotometer (ND-1000, Thermo Fisher Scientific Inc., Waltham, MA, USA) and high molecular weight DNA quality was verified by agarose gel electrophoresis. 2 *μ*g of genomic DNA was used for aCGH analysis following the manufacturer's protocol for the G4449A SurePrint G3 Human CGH 4 × 180 K Oligo Microarray Kit (each array contains 173,341 60 mer oligonucleotide probes, Agilent Technologies Inc., Santa Clara, CA, USA). The aCGH procedure was validated for prenatal testing using Agilent's DNA Analytical (version 4.0) with the built-in ADM-2 algorithm set at threshold value of 6, a cut off value of 0.25, and a filter of six continuous probes [2]. All base pair positions for detected genomic imbalances were designated according to the March 2006 Assembly (NCBI36/hg18) in the UCSC Human Genome browser (http://genome.ucsc.edu/).

**Figure 2:**
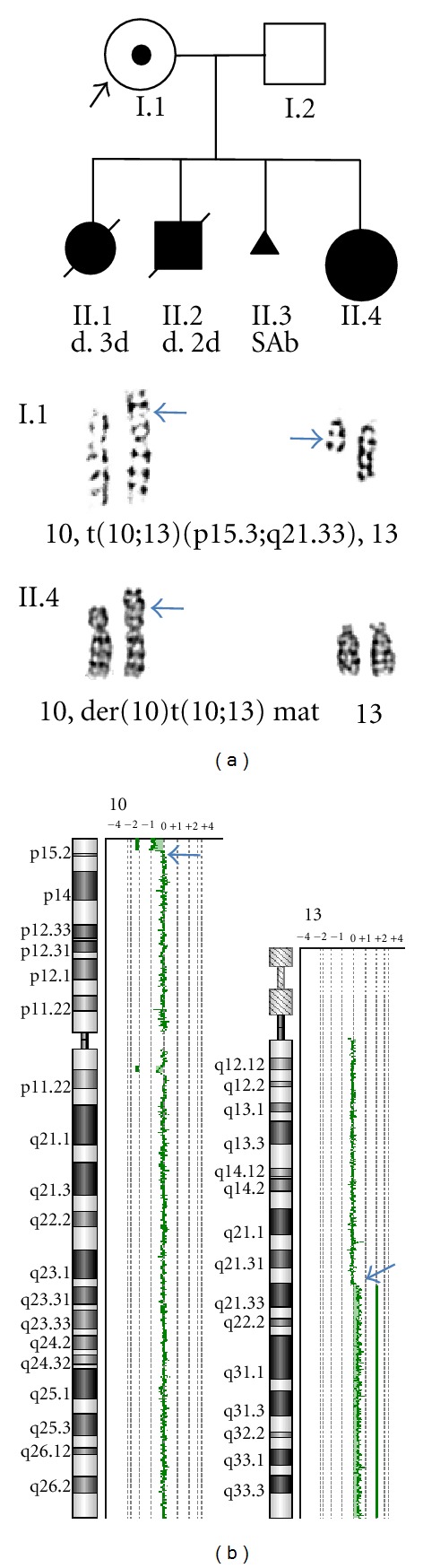
Cytogenomic results of the fetus. (a) Pedigree of the proband shows two neonatal deaths at 3 and 2 days (II.1 and II.2) and a spontaneous abortion (SAB, II.3). Arrows point to the reciprocal translocation of t(10;13) in the proband (I.1) and the derivative chromosome 10 in the fetus (II.4). (b) aCGH chromosome views show the small 2.339 Mb distal 10p deletion and the large 46.344 Mb 13q duplication (arrows point to the breakpoints).

The mother's karyotype was 46,XX,t(10;13)(p15.3;q21.33) and the fetus' karyotype from cultured amniocytes was 46,XX,der(10)t(10;13)(p15.3:q21.33)mat (Figure 2(a)). Oligonucleotide aCGH analysis revealed a 2.339 Mb deletion at 10p15 (chr10:126,161–2,465,089, containing refseq genes *ZMYND11*, *DIP2C*, *LARP4B*, *GTPBP14*, *IDI2*, *IDI2-AS1 *and *ADARB2*) and a 46.344 Mb duplication of 13q21.33–q34 (chr13:67,779,708–114,123,540, including 115 refseq genes from *KLHL1 *to *ZNF828*). The breakpoint at 10p15.3 is in a 20 Kb interval (chr10:2,465,089–2,485,482), and the breakpoint at 13q21.33 is in an 8.5 Kb interval (chr13:67,771,217–67,779,708). The breakpoints involve noncoding sequences, indicating that the translocation in the mother is truly balanced.

## 4. Discussion

To our knowledge, this is the first prenatal case of partial trisomy 13q and partial monosomy 10p detected by integrated cytogenetic and genomic analyses. There were three previously reported cases in the literature (Table 1). The first case showed partial monosomy 10p and partial trisomy 13q of a maternal carrier of a t(10;13)(p15;q22), and the phenotype was predominantly of partial trisomy 13q [3]. The second case showed pronounced features of 10p duplication from a double partial trisomy of 10p and 13q resulting from a 3 : 1 segregation of a maternal t(10;13)(p13;q22) [4]. The third case had de novo partial monosomy 10p (at least 4.8 Mb from 10pter) and partial trisomy 13q (estimated 0.7 Mb from 13qtel) from a der(10)t(10;13)(p15.2;q34), the congenital malformations might be associated with the partial 10p deletion and the craniofacial features might be attributed to the 13q duplication [5]. Partial trisomy 13q along with structural rearrangements of other chromosomes has been reviewed and those previously reported cases lacked the molecular mapping of breakpoints and gene content by the current genomic technology [6]. All of these previous observations indicated the importance of defining the breakpoints and gene content of compound abnormalities.

A two-step prenatal genetic counseling process has been applied to detect partial trisomy and partial monosomy from a parental carrier of a reciprocal translocation. The first step is the assessment of fetal viability through family history and pattern of the compound abnormalities. Unfortunately, chromosome analysis was not performed on the one early spontaneous abortion and two infants who expired at 2 or 3 days of age; therefore, there is no karyotype information for the underlying chromosomal abnormalities causing these severe conditions. Recently, an infant of partial trisomy 10p12.33 (19.5 Mb) and partial monosomy 13q32.1 (18.3 Mb) from a maternal t(10;13) showed intrauterine growth retardation, microphthalmia, macrocephaly, holoprosencephaly patent ductus arteriosus, renal agenesis imperforate anus, ambiguous genitalia, and vertebral anomaly and expired at 7 days of age [7]. Assuming that a large deletion will have more severe impact than a duplication in the similar size, the two neonatal deaths and one spontaneous abortion in our case may be caused by much larger partial monosomy 13q and smaller partial trisomy 10p. An empirical evaluation using combined partial trisomy/monosomy measured as % of haploid autosomal length (HAL) suggested that the 46.344 Mb duplication of 13q (1.7% of HAL) and the 2.339 Mb deletion of 10p (0.08% of HAL) in the fetus are within the maximum threshold of a viable imbalances of 3.72% HAL for partial trisomy and 3% HAL for partial monosomy from a maternal adjacent-1 segregation [8]. However, the accumulation of more cases with genomic characterization of compound partial trisomy and partial monosomy can be used to reevaluate the maximum threshold of viable imbalances using genomic measurements.

The second step is to predict the phenotype from the detected compound abnormalities, which could be challenging due to the limited information from the medical databases and the literature. A recent deletion map of chromosome 10p presents minimal regions of overlap (MRO) for mental retardation, language impairment, autism, and DiGeorge-syndrome 2 from 10p15.1 to 10p14 [9]. The 2.339 Mb deletion of 10p15.3 in the fetus is proximal to the MRO, and the clinical significance from this deletion is uncertain. A chromosome duplication map of chromosome 13 suggested the association of polydactyly, trigonocephaly, microphthalmia, cleft lip, inguinal hernia, umbilical hernia, and coloboma with partial trisomy of 13q21–q34 [10]. The fetus showed polydactyly by prenatal ultrasound examination and major features of distal trisomy 13 in postnatal examination. The clinical features from cases with compound abnormalities of partial monosomy or trisomy of 10p and partial trisomy of 13q are presented in Table 1.

In summary, genomic characterization of prenatally detected partial trisomy and partial monosomy has provided detailed information on unbalanced gene content and the breakpoints involving the balanced reciprocal translocation in the parental carrier. This information is helpful for prenatal genetic counseling of fetal viability and phenotype prediction. This cytogenomic approach should also be performed on recurrent spontaneous abortions, stillbirths, and neonatal deaths to reassess fetal viability thresholds using genomic information.

## Figures and Tables

**Table 1:** Phenotypes of partial 13q trisomy and partial 10p monosomy.

Cytogenomic findings, family history, and clinical features	Partial 13q trisomy	Partial 10p monosomy	First case Liu et al. 1986 [3]	Second case Yip et al. 1990 [4]	Third case Roos et al. 2006 [5]	This case
Karyotype			46,XX,der(10)	47,XX,t(10:13)(p13:q22),	46,XY,der(10)	46,XX,der(10)t(10;13)
			t(10;13)(p15;q22)mat	+der(13)t(10;13)mat	t(10;13)(p15.1;q34)	(p15.3:q21.33)mat

Genomic coordinate					D10S2488−,	(chr10:126,161–2,465,089)x1,
					D13S296+	(chr13:67,779,708–114,123,540)x3

Family history			Maternal t(10;13)	Maternal t(10;13)	De novo	Maternal t(10;13)
NL : CBT : SAB : ND : Affected*			(NA)	4 : 9 : 5 : 2 : 1	(NA)	0 : 0 : 1 : 2 : 1

Clinical features						
Psychomotor retardation	+	+	+	+	+	+
Low set ears	+	−	+	/	+	+
Hypertelorism	+	−	+	−		+
Thin upper lip	+	−	+	/	+	+
Long philtrum length	+	−	+	/	/	+
Wide depressed nasal bridge	+	−	+	/	/	+
High arch palate	+	−	+	/	+	+
Short broad neck	+	−	−	/	+	+
Cryptorchidism	+	−	−		−	−
Hemangioma	+	−	+	/	+	+
Polydactyly	+	−	+	/	−	+
Renal defect	−	+	−	/	−	−
Skeletal anomalies	−	+	+	+	−	−

*Ratio of cases in a pedigree. NL: normal; CBT: carrier of balanced translocation; SAB: spontaneous abortion; ND: newborn death. NA: not available.

## References

[1] Hall JG, Nelson WE (1996). C hromosomal clinical abnormalities. *Textbook of Pediatrics*.

[2] Li P, Pomianowski P, Dimaio MS (2011). Genomic characterization of prenatally detected chromosomal structural abnormalities using oligonucleotide array comparative genomic hybridization. *American Journal of Medical Genetics, Part A*.

[3] Liu XX, Yang ZR, Yu JW, Hu BX, Xu LH (1986). A case of distal partial trisomy of long arm in chromosome 13 resulting from the mother’s balanced translocation. *Journal of Tongji Medical University*.

[4] Yip MY, Williams J, Goddard A, Campbell P, Lambert I, Smithells RW (1990). Multiple abnormalities in a child with partial duplications of 10p and 13q from a 3:1 segregation of a maternal t(10;13) translocation. *Journal of Medical Genetics*.

[5] Roos A, Rudnik-Schöneborn S, Eggermann K (2006). Submicroscopic unbalanced translocation resulting indel10p/dup13q detected bysubtelomere FISH. *European Journal of Medical Genetics*.

[6] Machado IN, Heinrich JK, Campanhol C, Rodrigues-Peres RM, Oliveira FM, Barini R (2010). Prenatal diagnosis of a partial trisomy 13q (q14*⇢*qter): phenotype, cytogenetics and molecular characterization by spectral karyotyping and array comparative genomic hybridization. *Genetics and Molecular Research*.

[7] Puvabanditsin S, Garrow E, Lambert G (2011). Partial trisomy 10p12.33 and partial monosomy 13q32.1: case report and a literature review. *Genetic Counseling*.

[8] Cohen O, Cans C, Mermet MA, Demongeot J, Jalbert P (1994). Viability thresholds for partial trisomies and monosomies: a study of 1,159 viable unbalanced reciprocal translocations. *Human Genetics*.

[9] Lindstrand A, Malmgren H, Verri A (2010). Molecular and clinical characterization of patients with overlapping 10p deletions. *American Journal of Medical Genetics, Part A*.

[10] Brewer C, Holloway S, Zawalnyski P, Schinzel A, FitzPatrick D (1999). A chromosomal duplication map of malformations: regions of suspected haplo- and triplolethality—and tolerance of segmental aneuploidy—in humans. *American Journal of Human Genetics*.

